# DNA watermarks in non-coding regulatory sequences

**DOI:** 10.1186/1756-0500-2-125

**Published:** 2009-07-07

**Authors:** Dominik Heider, Martin Pyka, Angelika Barnekow

**Affiliations:** 1 Department of Experimental Tumorbiology, University of Münster, Badestr. 9, D-48149 Münster, Germany; 2 Interdisciplinary Center for Clinical Research, University Hospital Münster, Domagkstr. 3, D-48143 Münster, Germany; 3 Department of Bioinformatics, Center for Medical Biotechnology, University of Duisburg-Essen, Universitätsstr. 2, D-45117 Essen, Germany

## Abstract

**Background:**

DNA watermarks can be applied to identify the unauthorized use of genetically modified organisms. It has been shown that coding regions can be used to encrypt information into living organisms by using the DNA-Crypt algorithm. Yet, if the sequence of interest presents a non-coding DNA sequence, either the function of a resulting functional RNA molecule or a regulatory sequence, such as a promoter, could be affected. For our studies we used the small cytoplasmic RNA 1 in yeast and the lac promoter region of *Escherichia coli*.

**Findings:**

The lac promoter was deactivated by the integrated watermark. In addition, the RNA molecules displayed altered configurations after introducing a watermark, but surprisingly were functionally intact, which has been verified by analyzing the growth characteristics of both wild type and watermarked scR1 transformed yeast cells. In a third approach we introduced a second overlapping watermark into the lac promoter, which did not affect the promoter activity.

**Conclusion:**

Even though the watermarked RNA and one of the watermarked promoters did not show any significant differences compared to the wild type RNA and wild type promoter region, respectively, it cannot be generalized that other RNA molecules or regulatory sequences behave accordingly. Therefore, we do not recommend integrating watermark sequences into regulatory regions.

## Background

DNA watermarks can be used for authenticating genetically modified organisms or in future for labeling animals in breeding [1,2]. It has already been shown *in silico *and *in vivo *that these watermarks do not affect the translation of proteins [1-4]. These assumptions only apply to coding regions, thus the insertion of watermarks into regulatory sequences like promoter regions or regulatory RNA molecules, had to be examined. In our studies we integrated watermark sequences into a widely known promoter region of bacteria. Watermarks integrated into regulatory regions like promoter or enhancer sequences can affect their functionality. We integrated watermark sequences into the lac promoter of *Escherichia coli *to examine, whether the watermarks affect the promoter activity. The lac operon of *Escherichia coli *consists of a promoter, three operators and three genes (lacY, lacZ and lacA), coding for the *β*-galactoside permease, *β*-galactosidase and *β*-galactoside transacetylase, which are required for the lactose metabolism in *Escherichia coli *[5]. The *β*-galactosidase cleaves the lactose into glucose and galactose [5]. The promoter sequence of the lac operon consists of two highly conserved regions called the -35 (TTTACA) and -10 (TATGTT) regions, upstream from the transcription start. It has already been shown that single point mutations within conserved regions can affect or even stop promoter activity of different promoters (Additional file 1) [6-10].

In a second approach we integrated a watermark sequence into a regulatory RNA molecule called small cytoplasmic RNA 1 (scR1) from yeast (scR1 gene on chromosome V of *Saccharomyces cerevisiae*, ). The scR1 consists of 519 nucleotides and represents the homologous RNA molecule in *Saccharomyces cerevisiae *to the mammalian 7SL RNA, which is part of the signal recognition particle (SRP), necessary for the targeting of proteins into the endoplasmic reticulum (ER) [11-13]. The SRP complex is a ribonucleoprotein consisting of six polypeptides and the 7SL RNA. In *Saccharomyces cerevisiae *the six polypeptides are SRP14, SRP19, SRP21, SRP54, SRP68 and SRP72 [14]. The SRP displays a GTPase activity [15]. The 7SL molecule is a conserved functional RNA constituting the backbone of the SRP complex and has been shown to be essential for all organisms tested, except *Saccharomyces cerevisiae *cells. *Saccharomyces cerevisiae *cells are viable without scR1, but have a reduced reproduction rate [11,12,14,16-21].

## Results and discussion

The analyses of the watermarked DNA sequences of both, the scR1 and the lac promoter sequence with the DNA-Crypt fuzzy controller, reveal that the use of any correction code is not recommended.

We inserted a watermark sequence starting at position 898 in the lac promoter of pBluescriptII KS+ plasmid. The watermark sequence deactivated the lac promoter, which was verified by qualitative *β*-galactosidase assay using X-Gal (5-bromo-4-chloro-3-indolyl-*β*-D-galactopyranoside) as a substrate (Table 1).

**Table 1:** Qualitative *β*-galactosidase assays

promoter	qualitative *β*-galactosidase assay
lac	+

lac K5	+

lac II	-

lac wt: the wild type lac promoter

lac K5: the lac promoter containing the watermark sequence WWU

lac II: the lac promoter containing the watermark sequence 42

+: promoter is active

-: promoter is inactive

**Figure 1:**
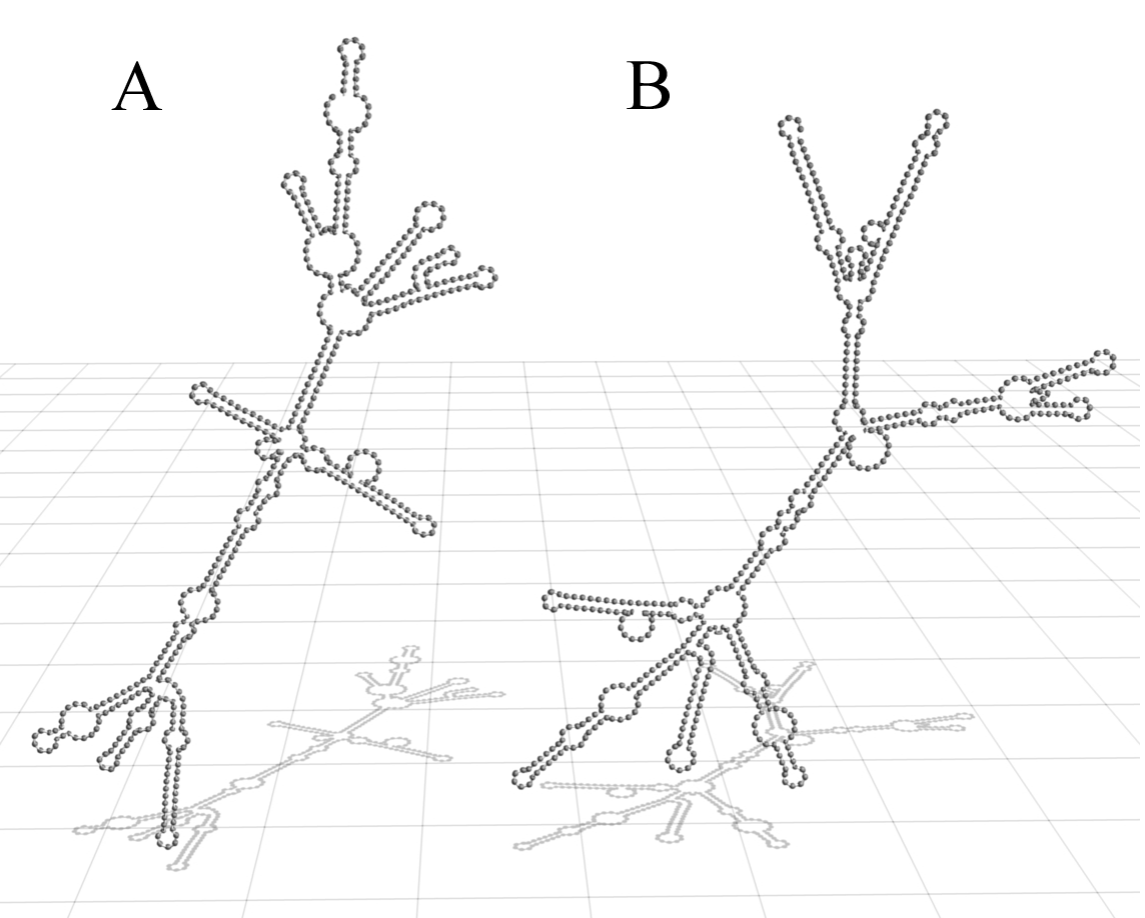
**Secondary structure of the wild type scR1 and the watermarked scR1**. The secondary structure predictions were performed using the ViennaRNA-1.5 web interface. The 3D model was created using Blender. A: wild type scR1 of *Saccharomyces cerevisiae*; B: watermarked scR1 (see section methods)

In 2001, Dieci et al. showed that after transformation of YRA130 ΔscR1 yeast cells with the wild type scR1 Yep352 plasmid, a normal division rate was achieved and the wild type phenotype could be rescued [12]. For our studies we transformed the YRA130 ΔscR1 yeast cells with the Yep352 plasmid containing a watermarked scR1 gene (Yep352-SCR1-TB). The watermark sequence was integrated into the wild type scR1 gene starting at position 471 (see mutagenic primer sequences). The secondary structure predictions of the watermarked scR1 using the ViennaRNA-1.5 web interface revealed significant changes within the secondary structure compared to the structure of the wild type scR1 (Figure 1) [22]. The 3D model was created using Blender .

**Figure 2:**
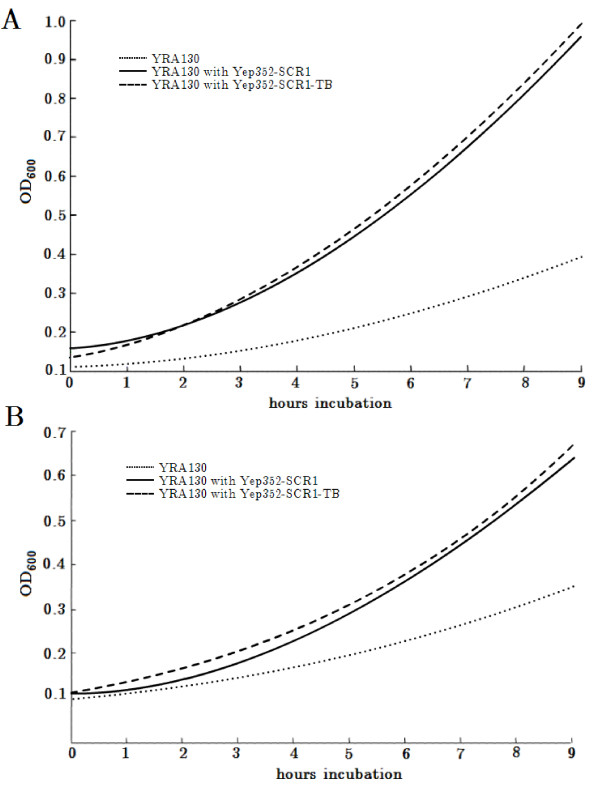
**Growth characteristics**. Growth characteristics of strain YRA130 and the Yep352-SCR1 and Yep352-SCR1-TB transformed YRA130 strains in YPD full medium (A) and SD-ura medium (B). Three independent experiments with a standard deviation <0.03 are shown. All data show p-values <0.05, except YRA130 cells transformed with Yep352-SCR1 compared with YRA130 cells transformed with Yep352-SCR1-TB.

Although the predicted secondary structure of the watermarked scR1 differs from the wild type structure, the resulting RNA molecule proved to be functional active indirectly demonstrated by the YRA130 cells transformed either with the Yep352-SCR1 or the Yep352-SCR1-TB, which displayed no significant differences in their growth characteristics (Figure 2).

**Figure 3:**
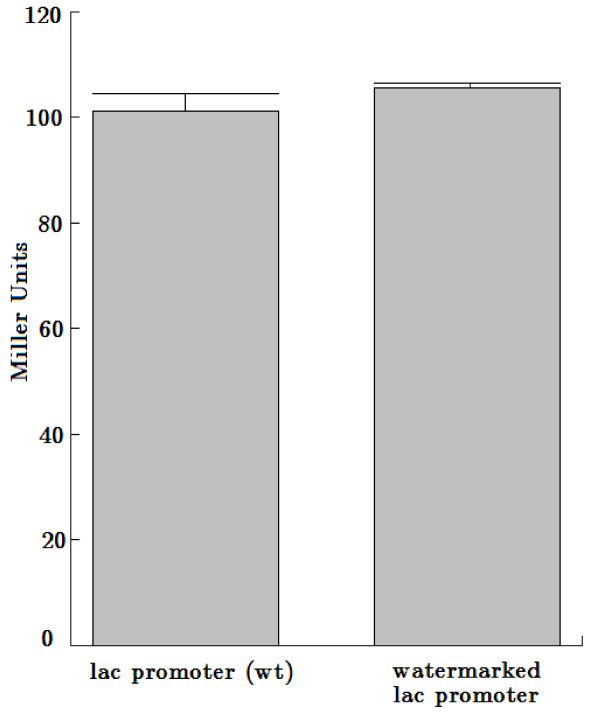
**Quantitative *β*-galactosidase assay with ONPG**. Three independent analyses were performed.

In addition, we inserted a second overlapping watermark sequence into the lac promoter region. The watermark sequence was integrated into the wild type lac promoter sequence starting at position 903 in the pBluescriptII KS+ plasmid and overlapped with the aforementioned deactivating watermark sequence. The watermarked lac promoter was proved to be active, verified by the qualitative *β*-galactosidase assay using X-Gal. Furthermore, the watermarked lac promoter did not display any significant differences compared to the wild type lac promoter using the quantitative *β*-galactosidase assay with ONPG (ortho-Nitrophenyl-*β*-galactoside) (Table 1, Figure 3).

## Conclusion

Our results show that the integration of watermarks is not suitable for regulatory regions like promoter or regulatory RNA molecules. We have shown that these watermarks can deactivate promoter regions and further affect the secondary structure of regulatory RNA molecules. Although the watermarked scR1 was not deactivated by the integration of a watermark sequence, the secondary structure prediction displayed an altered structure. These results cannot be generalized for other RNA molecules.

The affect of integrated watermarks in non-coding regulatory regions cannot be generalized and has to be tested in every single case. Therefore, we propose not to integrate watermark sequences into regulatory sequences.

## Methods

### Watermark design

We wanted to integrate a watermark into the lac promoter containing the answer to life, the universe, and everything [23]. Further we wanted to encrypt the watermark 'TB' into the scR1 gene and 'WWU' into the lac promoter sequence, respectively. To demonstrate the flexibility of our algorithm we used different translation codes. The first one only used the binary representation. The second translation code used for the scR1 experiments just slightly differs from the standard one used in DNA-Crypt [1]. The third one imitates the official international Morse code. But identical binary encoding tables described in Heider and Barnekow 2007 were used [1]. The inserted DNA watermark sequences are

42 → 101010_2 _→ CCC

TB → 1001100001_2 _→ CGCTG

and

WWU → .--.--..- → 0110110011_2 _→ GCATA

We created artificial reading frames within the scR1 gene and the lac promoter sequence that do not exist in non-coding regions, to apply the DNA-Crypt algorithm. Because of cost-benefit equation, we scanned manually for the best location within the DNA sequences of the scR1 gene and the lac promoter for integrating authenticating watermarks. The two watermark sequences in the lac promoter region overlap.

### DNA-Crypt fuzzy controller

The watermark sequences were analyzed with the DNA-Crypt fuzzy controller with standard settings. The life time was set at 1000 cycles [1]. Based on the three input dimensions, the length of the watermark sequence, individual mutation rate and life time, the DNA-Crypt fuzzy controller recommends, based on the rule base, whether to use a specific mutation correction code or not [1].

### Secondary structure prediction

The secondary structure predictions of the RNA molecules were performed using the ViennaRNA-1.5 web interface with rescaled energy parameters to 30°C [22]. The three dimensional structure predictions were created with Blender.

### Site-directed mutagenesis

The site-directed mutagenesis was used to integrate the watermark sequences into the wild type DNA. We used a modified QuikChange(R) Site-Directed Mutagenesis Kit protocol from Stratagene (Stratagene, Amsterdam, The Netherlands) described in Heider and Barnekow 2008 [4]. For the scR1 studies we used the Yep352-SCR1 plasmid carrying the wild type sequence of the scR1 gene (a kind gift of Giorgio Dieci, Universita di Parma, Italy) [12]. For our lac promoter studies we used the pBluescriptII KS+ plasmid (Stratagene, Amsterdam, The Netherlands). The site-directed mutagenesis was performed with the following primer sequences:

scR1 – forward primer:

5'-GCACATTGTGGC**C**GT**G**CC**C**TC**T**GG**G**ATGGAGTGTGTC-3'

scR1 – reverse primer:

5'-GACACACTCCATCCCAGAGGGCACGGCCACAATGTGC-3'

lac – forward primer:

5'-GTTAGCTCACTCATTTGGGACCCCAGGCTTTACA-3'

lac – reverse primer:

5'-TGTAA**A**GC**C**TGGG**G**TCCCAAATGAGTGAGCTAAC-3'

lac2 – forward primer:

5'-CTCACTCATTAGGC**C**CC**C**CAGGC**C**TTACACTTTATGC-3'

lac2 – reverse primer:

5'-GCATAAAGTGTAAGGCCTGGGGGGCCTAATGAGTGAG-3'

The bold letters represent the watermark sequences. The mutagenesis was confirmed by sequencing with the M13 primer 5'-GTAAAACGACGGCCAGT-3' and the lac-SEQ primer 5'-CCGCCTCTCCCCGCG-3', respectively.

### Transformation of bacteria

For our lac promoter studies we used the *Escherichia coli *DH5*α *strain. 20 ml 2YT were inoculated with 200 *μ*l overnight culture of DH5*α *bacteria and incubated at 37°C to an OD_600 _of 0.3 to 0.5. The cells were centrifuged for 10 minutes at 2000 × g and mixed with 10 ml ice-cold sterile 0.1 M CaCl_2_. After incubation for 30 minutes on ice, the cells were centrifuged for 10 minutes at 4°C 2000 × g and resuspended in 2 ml ice-cold 0.1 M CaCl_2_. 200 *μ*l of the cells were mixed up with 50 ng DNA. After incubation for 30 minutes on ice, the cells were incubated for 45 seconds at 42°C and cooled down for 2 minutes on ice. After mixing with 500 *μ*l 2YT the cells were incubated for 45 minutes at 37°C and 220 rpm. Finally 200 *μ*l of the cell suspension were plated and grown overnight on 2YT plates containing ampicillin.

### Transformation of yeast

We used the YRA130 ΔscR1::His3 yeast strain for our scR1 experiments that has a reduced reproduction rate (a kind gift of Peter Walter, University of California, San Francisco, USA). The yeast strain YRA130 was transformed using the lithium acetate method and grown on SD-ura plates [24].

### *β*-galactosidase assay

The activity of the lac promoter was verified by qualitative and quantitative *β*-galactosidase assays, using 5-bromo-4-chloro-3-indolyl-*β*-D-galactopyranoside (X-Gal, Carl Roth GmbH, Karlsruhe, Germany) and ONPG (Carl Roth GmbH, Karlsruhe, Germany), respectively. For the qualitative *β*-galactosidase assay we pipetted 40 *μ*l 100 mM IPTG (Carl Roth GmbH, Karlsruhe, Germany) and 40 *μ*l X-Gal (20 mg/ml) onto a 2YT agar plate containing ampicillin. After drying the plates for one hour, 20 *μ*l of overnight cultures were plated and incubated overnight at 37°C. The quantitative analyses we performed using the method established by Miller in 1972, except some modifications published by Zhang and Bremer and some additional modifications [25,26]. We used 100 *μ*l of IPTG induced overnight culture for our assays and froze them for 30 minutes at 20°C before adding the permealization buffer. In addition we used a substrate buffer containing 3 mg/ml instead of 1 mg/ml ONPG to guarantee saturation for the *β*-galactosidase.

### Growth characteristics

The growth characteristics of YRA130, and the Yep352-SCR1 and Yep352-SCR1-TB transformed YRA130 cells were analyzed by measuring optical densities at 600 nm every 60 minutes for nine hours (Pharmacia LKB Novaspec II).

## Competing interests

The authors declare that they have no competing interests.

## Authors' contributions

DH: conception, structure predictions, sequence alignments, mutagenesis, transformation of yeast and bacteria cells, microscopy, *β*-galactosidase assays, growth characteristics, figure preparation, manuscript preparation. MP: secondary structure visualization. AB: conception, manuscript preparation. All authors read and approved the final manuscript.

## Supplementary Material

Additional file 1 **Table S1 – Conserved promoter regions**. lac: the lac promoter; hexokinase: Rat type III hexokinase promoter; H2B: histone H2B promoter; AD4: adenovirus type 4; AD2: adenovirus type 2; SV40: SV40 enhancer Click here for file
