# Supplementary material for: DNA watermarks in non-coding regulatory sequences
Source: BMC Res Notes. 2009 Jul 7;2:125. doi: 10.1186/1756-0500-2-125 (PMC2713970; doi:10.1186/1756-0500-2-125)
Supplement: Additional file 1 — Table S1 – Conserved promoter regions. lac: the lac promoter; hexokinase: Rat type III hexokinase promoter; H2B: histone H2B promoter; AD4: adenovirus type 4; AD2: adenovirus type 2; SV40: SV40 enhancer [file 1756-0500-2-125-S1.pdf]

Table 1

promoter	***sequence***
lac	TAGCTCACTCATTAGGCACCCCAGGC**TTTACA**CTTTATGCTTCCGGCTCG**TATGTT**GTGTGGAAT
hexokinase	AAT**TAGCATAT**TTCTGTTGAGTGGCTGDDGAGGAGGGTGGAGCTTGGCCCTGAGC**TTCACTTCT**G
AD4	TTTTTTTTGTGTGAG**TTAATATGCAAATAAGG**CGTGAAAATTTGGGGAT
H2B	GTCGGTCCGCTGCGA**T**GCG**TATGCAAAT**GTTAGCCATTCAGGGGGGATT
AD2	TATTTTGGATTGAAGCCA**ATATG**AT**AAT**G**AGG**GGGGGTGGAGTTTGTGACG
SV40	GCTCCCCAGCAGGCAGA**A**G**TATGCAAA**GC**A**T**G**CATCTCAATTAGTCAGC
